# Altern und Sterben im Strafvollzug

**DOI:** 10.1007/s00103-025-04135-0

**Published:** 2025-10-22

**Authors:** Michael Lindemann, Torsten Verrel

**Affiliations:** 1 Lehrstuhl für Strafrecht, Strafprozessrecht und Kriminologie, Fakultät für Rechtswissenschaft, Universität Bielefeld, Postfach 10 01 31, 33501 Bielefeld, Deutschland; 2 Institut für Medizinstrafrecht und Kriminologisches Seminar, Rechts- und Staatswissenschaftliche Fakultät, Universität Bonn, Adenauer Allee 24–42, 53113 Bonn, Deutschland

**Keywords:** Strafvollzug, Lebensältere Gefangene, Sterben und Tod, Suizid, Altersmedizin, Prison system, Elderly prisoners, Dying and death, Suicide, Geriatric medicine

## Abstract

Die Überalterung der Gesellschaft führt auch im Strafvollzug zu einer wachsenden Zahl lebensälterer Gefangener. Die damit verbundenen Herausforderungen beziehen sich nicht nur auf eine den gesetzlichen Anforderungen entsprechende Behandlung und medizinische Versorgung von Gefangenen mit der Perspektive einer Haftentlassung. Der Strafvollzug muss gleichermaßen einen angemessenen Umgang mit solchen Gefangenen finden, die ihr Lebensende in einer Justizvollzugsanstalt verbringen. Darauf ist der Strafvollzug bislang nur ansatzweise eingestellt. Dies betrifft in erster Linie die sachlichen und personellen Anforderungen an einen altengerechten Strafvollzug. Gefangene sollten allerdings möglichst nicht im Gefängnis sterben müssen und es bestehen auch rechtliche Möglichkeiten, die den Weg zu einem Sterben in Freiheit eröffnen. Kommt eine Entlassung zum Lebensende gleichwohl nicht in Betracht, müssen passende Rahmenbedingungen für das würdige alters- oder krankheitsbedingte Sterben im Vollzug geschaffen werden. In rechtlicher Hinsicht hat das Urteil des Bundesverfassungsgerichts zum Grundrecht auf selbstbestimmtes Sterben die Frage nach sich gezogen, ob sterbewilligen Gefangenen der Zugang zu Suizidmitteln ermöglicht werden muss. Obschon dies nicht kategorisch ausgeschlossen werden kann, muss Besonderheiten der Vollzugssituation – etwa durch eine besonders sorgfältige Prüfung der Freiverantwortlichkeit des Suizidentschlusses – Rechnung getragen werden.

## Abstract (en)

The aging of society is also leading to a growing number of elderly prisoners in the prison system. The challenges associated with this relate not only to the treatment and medical care of prisoners in accordance with legal requirements with the prospect of release from prison. The prison system must also find an appropriate way of dealing with prisoners who are spending the end of their lives in a correctional facility. So far, the prison system has only begun to address this issue. This primarily concerns the material and personnel requirements for a prison system that is suitable for the elderly. If possible, prisoners should not have to die in prison, and there are also legal options that open the possibility of dying in liberty. However, if release at the end of life is not an option, appropriate conditions must be created for dignified age- or illness-related dying in prison. From a legal perspective, the judgement of the Federal Constitutional Court on the fundamental right to a self-determined death has raised the question of whether prisoners who wish to die must be given access to means of suicide. Although this cannot be categorically ruled out, the special features of the prison situation must be taken into account—for example, through a particularly careful examination of whether the decision to commit suicide was made freely and responsibly.

## Einleitung

Alte, gar dem Tode nahe Gefangene entsprechen nicht der allgemeinen Vorstellung von der Strafvollzugspopulation. In Gefängnissen – das Gesetz spricht von Justizvollzugsanstalten (JVA)^1^ – erwarten wir junge, allenfalls mittelalte Männer,^2^ die wegen erheblicher (Gewalt‑)Straftaten verurteilt wurden.

**Abb. 1:**
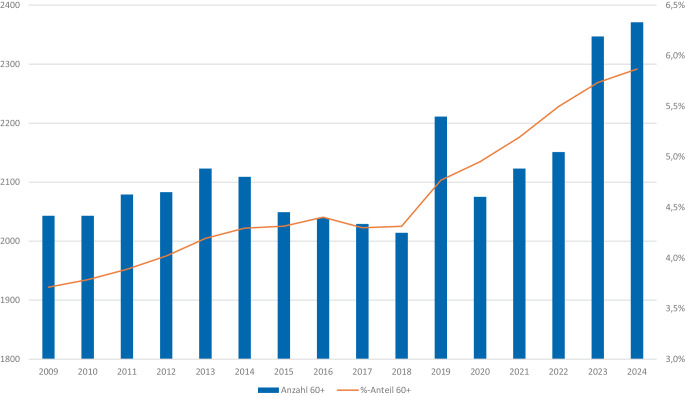
Strafgefangene mit Freiheitsstrafe der Altersgruppe 60 Jahre und älter, Deutschland 2009–2024. (Quelle: eigene Auswertung vom Statistischen Bundesamt, 2009–2021: Fachserie 10 Reihe 4.1, Tab. 2, seit 2023 Statistischer Bericht Strafvollzug, Tab. 24321–03)

Inhaftierte im Rentenalter können aber beispielsweise Personen sein, die als Spätkriminelle erstmals durch erhebliche Straftaten, wie etwa Kapitaldelikte in Nähebeziehungen oder schwerwiegende Vermögensdelikte, auffällig geworden sind. Hinzu kommen solche Gefangenen, die bereits eine (lebens)lange kriminelle Karriere mit unter Umständen schon mehrjährigen Gefängnisaufenthalten aufweisen, es also nicht geschafft haben, in früherem Alter einen Ausstieg aus Kriminalität und Inhaftierung zu finden. Zwar handelt es sich bei den sogenannten lebensälteren Strafgefangenen, für die üblicherweise die Altersgruppe „60 plus“ gebildet wird, noch um eine Minderheit mit einem Anteil von fast 6 % an allen Gefangenen, die eine Freiheitsstrafe verbüßen (Stand 2024). Wie die aus Abb. 1 ersichtliche Entwicklung der letzten 15 Jahre zeigt, nimmt dieser Anteil jedoch stetig und mit einem besonders deutlichen Anstieg seit 2018 zu.

**Abb. 2:**
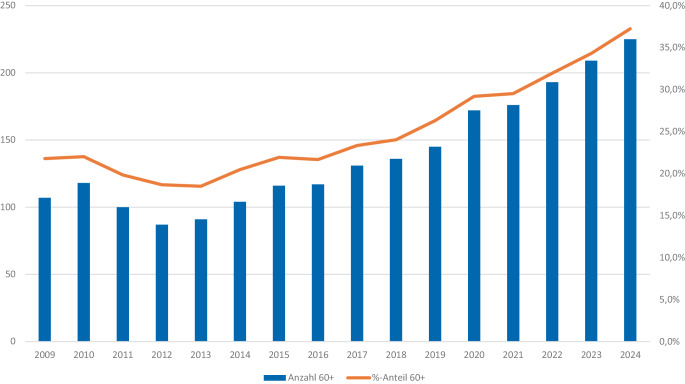
Sicherungsverwahrte der Altersgruppe 60 Jahre und älter, Deutschland 2009–2024. (Quelle: eigene Auswertung vom Statistischen Bundesamt, 2009–2021: Fachserie 10 Reihe 4.1, Tab. 2, seit 2023 Statistischer Bericht Strafvollzug, Tab. 24321-03)

Ausgeprägter ist die Situation im Vollzug der Sicherungsverwahrung, in der Personen im Anschluss an die Strafhaft allein aufgrund ihrer Gefährlichkeit unbefristet untergebracht werden (§§ 66 ff. Strafgesetzbuch, StGB). Dort entfielen auf die hier in Rede stehende Altersgruppe im Jahr 2024 gut 37 % aller Verwahrten, womit sich deren Anteil seit 2013 verdoppelt hat (Abb. 2). Das ist umso bemerkenswerter, als die Neuordnung der Sicherungsverwahrung im Jahr 2012 das Ziel hatte, die Anordnung und Dauer von Sicherungsverwahrungen spürbar zu begrenzen.

Die beschriebene Entwicklung ist nicht Ausdruck einer in Anzahl und Schwere zunehmenden Altersdelinquenz. Vielmehr ist die kriminelle Belastung dieser Altersgruppe seit jeher niedrig und im Längsschnitt tendenziell sogar rückläufig [2, 3]. Die sowohl absolut als auch relativ wachsende Zahl älterer Gefangener ist vor allem Ausdruck des demografischen Wandels, der nicht nur in deutschen Gefängnissen zu beobachten ist [4]. Ältere begehen also nicht mehr oder schwerere Straftaten als früher, vielmehr führt die Alterung der Gesellschaft zu mehr potenziellen Tätern in fortgeschrittenem Alter. Ob die weiter zunehmende Lebenserwartung und die damit voraussichtlich verbundene Zunahme von Tatanreizen und -gelegenheiten zu einer echten Kriminalitätssteigerung führen, bleibt abzuwarten.

In der Literatur wird weiter angenommen, dass die seit Ende der 1990er-Jahre zu beobachtende Verschärfung der Sanktionspraxis zu längeren Verweildauern geführt hat [5–7]. Unabhängig davon, ob diese Einschätzung geteilt wird, genügt an dieser Stelle der Befund einer stetig steigenden Zahl lebensälterer Strafgefangener, der zu der Frage führt, wie es um das Altern und womöglich sogar Sterben im Strafvollzug bestellt ist.

Die im Folgenden gegebenen Antworten beschränken sich auf juristisch-kriminologische Aspekte. Zunächst wird auf die Probleme eingegangen, die sich im Vollzugsalltag im Umgang mit lebensälteren Strafgefangenen ergeben. Danach und im Schwerpunkt sollen die Herausforderungen angesprochen werden, die mit einem Lebensende im Strafvollzug, also dem Sterben im Gefängnis, verbunden sind.

## Altern im Strafvollzug

Wenn gesagt wird, dass der Strafvollzug ein Spiegel der Gesellschaft ist [8], so trifft das nicht nur auf die in den Gefängnissen angekommene Überalterung der Gesellschaft, sondern auch auf den wachsenden Bedarf zu, altengerechte Lebens- und Umgebungsverhältnisse zu schaffen. Die Deckung dieses Bedarfs durch den Strafvollzug ist freilich mit besonderen Schwierigkeiten verbunden. Im Unterschied zum Altern in Freiheit liegt die Verantwortung für das Altern im Vollzug allein in staatlicher Hand, denn Gefangene können insoweit keine private Vorsorge betreiben und ihre Haftbedingungen nicht beeinflussen. Diese Bedingungen zeichnen sich dadurch aus, dass Menschen mit oftmals erheblichen Sozialisationsdefiziten und daraus resultierenden Gefahren für die Anstaltssicherheit und -ordnung in einer abgeschlossenen und durchregulierten, damit potenziell zu Unselbstständigkeit führenden Zwangsgemeinschaft leben. Die Gestaltungsmöglichkeiten für einen Altenstrafvollzug werden dadurch von vornherein eingeschränkt.

Hinzu kommt, dass viele Gefangene aus unterschiedlichen Gründen vorgealtert sind [9–11], weshalb sich schon bei deutlich jüngeren Personen als außerhalb des Vollzugs der Bedarf für eine altersgerechte Versorgung und Betreuung ergeben kann. Aus diesem Grund erscheint die Vollendung des 60. Lebensjahres als Grenze für die Zuweisung zur Gruppe „alter“ Gefangener als sachgerecht. Allerding bestehen durchaus Bedenken, allgemein von „Alterskriminalität“ schon bei so „jungen“ Tätern zu sprechen, da sich das Ende der Berufstätigkeit immer weiter nach hinten verlagert und die körperliche und geistige Fitness länger anhält. Schließlich ist als weitere Erschwernis die hohe Prävalenz psychischer Störungen unter Gefangenen zu nennen, die zu den ohnehin bestehenden „normalen“ psychischen Belastungen der Haftsituation hinzutritt [9, 10].

## Rechtsrahmen

Die sich daraus ergebenden Herausforderungen und Probleme sind nicht primär rechtlicher Natur, sondern haben vor allem mit der sachlichen und personellen Ausstattung eines Strafvollzugs zu tun, der herkömmlich auf jüngere Gefangene und nicht auf ein „successful aging“ [10] und pflegebedürftige Gefangene ausgerichtet ist. So gibt es zwar keine spezifischen Vorschriften für lebensältere Strafgefangene, doch folgt aus dem allgemeinen Gegensteuerungsgrundsatz die Verpflichtung, gerade vulnerable Gefangene wie die Lebensälteren vor subkulturellen Gefahren, insbesondere vor Gewaltanwendung und -androhung, zu schützen (zur Gewalt unter Gefangenen Neubacher [5]) und einer vollzugsbedingten Beschleunigung von Abbauprozessen entgegenzutreten [9]. Normiert ist der Gegensteuerungsgrundsatz beispielsweise in § 2 Abs. 1 Satz 4 des Strafvollzugsgesetzes Nordrhein-Westfalen (StVollzG NRW), auf das hier und im Weiteren stellvertretend für die anderen Länderstrafvollzugsgesetze verwiesen wird.

Im Angleichungsgrundsatz (§ 2 Abs. 1 Satz 1 StVollzG NRW) sowie auch in Nr. 102.2 der Europäischen Strafvollzugsgrundsätze^3^ ist das Prinzip verankert, dass die mit dem Freiheitsentzug zwangsläufig verbundene Strafwirkung nicht noch durch die Haftbedingungen verstärkt werden darf. Dies gebietet eine altengerechte Ausstattung der Hafträume, einschließlich der sanitären Anlagen, und einen barrierefreien Zugang zu Gemeinschaftseinrichtungen. Hinzu kommt, dass vor allem der allgemeine Vollzugsdienst, der den intensivsten Gefangenenkontakt aufweist, daneben aber auch der soziale und der psychologische Dienst auf die besonderen Bedürfnisse lebensälterer Gefangener vorbereitet sein sollten [9].

Die Gewährleistung einer äquivalenten geriatrischen Versorgung – mit Ausnahme der freien Arzt‑/Ärztinnenwahl – ergibt sich aus der in allen Strafvollzugsgesetzen mit unterschiedlichen Formulierungen normierten Verpflichtung der Vollzugsbehörden, für das „körperliche, seelische, geistige und soziale Wohlergehen der Gefangenen zu sorgen“ (§ 43 Abs. 1 Satz 1 StVollzG NRW). Dies beinhaltet nicht nur eine medizinische Behandlung, die dem Angebot der gesetzlichen Krankenversicherungen entspricht und Untersuchungen zur Früherkennung einschließt, sondern auch die Versorgung mit Hilfsmitteln (§ 45 Abs. 1 StVollzG NRW), wie beispielsweise Rollstühlen.

Sollten die erforderliche medizinische Behandlung und Betreuung in der für den lebensälteren Gefangenen zuständigen Vollzugsanstalt nicht möglich sein, kommt nach allgemeinen Regeln eine (vorübergehende) Überstellung oder (dauerhafte) Verlegung in eine dafür besser geeignete Vollzugseinrichtung in Betracht. Dabei kann es sich namentlich um ein Justizvollzugskrankenhaus (JVK), alternativ aber auch um eine externe Einrichtung handeln, in der dann eine ambulante oder ggf. auch stationäre Versorgung erfolgt (§ 46 StVollzG NRW).

Aus dem Vollzugziel, den Gefangenen für ein straffreies Leben in Freiheit zu ertüchtigen (§ 1 Satz 1 StVollzG NRW), kann für lebensältere Gefangene mit einer Entlassungsperspektive abgeleitet werden, dass sie auf altersspezifische Probleme der Wiedereingliederung, wie etwa die Suche nach altersgerechtem Wohnraum oder die Herausforderungen der Digitalisierung, vorbereitet werden müssen, mithin ein alterssensibles Übergangsmanagement zu etablieren ist [10].

## Istzustand

Es ist nun keineswegs so, dass der soeben skizzierte Sollzustand eines altengerechten Strafvollzugs keinerlei Entsprechung in der Strafvollzugswirklichkeit gefunden hat. Vielmehr befassen sich Strafvollzugspraxis und -wissenschaft schon lange mit den Konsequenzen des demografischen Wandels und es gibt eine beachtliche Zahl von Angeboten für ältere Inhaftierte (einen Überblick über die Situation im Vollzug der Sicherungsverwahrung geben Leuschner und Rausch [9], über die Ergebnisse einer 2014 erfolgten Abfrage berichten Berger-Zell [12, S. 368 f.]).

Dazu zählen etwa Maßnahmen zum Erhalt der auch kognitiven Gesundheit, Beschäftigungs‑, Bildungs- und Freizeitangebote sowie die Vermittlung von Alltags- und Sozialkompetenzen in Gesprächsgruppen und durch Mitgestaltungsmöglichkeiten [10, 13]. Dennoch ist unübersehbar, dass es sich bislang um einen Flickenteppich handelt und es weitgehend der Initiative und dem Engagement des Vollzugsstabs überlassen bleibt, ob und in welchem Umfang altersspezifische Angebote gemacht werden. Ein einheitlicher Standard für die Ausgestaltung eines Altenstrafvollzugs besteht damit nicht [10].

Konzeptionell können 2 unterschiedliche Wege beschritten werden. Da es sich noch um eine kleine Zahl von Strafgefangenen handelt, die Herstellung altersgerechter baulicher Verhältnisse kostenintensiv ist und die Gewinnung und Ausbildung qualifizierten Personals erhebliche Anstrengungen erfordern, scheint eine zentrale Einrichtung besonderer Anstalten oder Abteilungen für Altenstrafvollzug sinnvoll zu sein, die zudem Distanz zur Subkultur jüngerer Gefangener schafft. Diese Form der von den Strafvollzugsgesetzen geforderten Behandlungsbinnendifferenzierung (§ 93 StVollzG NRW) wurde etwa in der Lebensälterenabteilung der JVA Bielefeld-Senne mit der in Nordrhein-Westfalen höchsten Zahl von 87 Haftplätzen realisiert [14].

Für dezentrale, d. h. in bestehende Anstalten integrierte besondere Haftplätze spricht dagegen die Ermöglichung eines heimatnahen Vollzugs und auch, dass Kontakte zu jüngeren Strafgefangenen für beide Seiten einen Nutzen haben können [9].

Vorzugswürdig dürfte eine Mischung beider Anstaltsorganisationsformen sein (näher zu den Vor- und Nachteilen Ghanem et al. [4, S. 372 ff.]), mit der den unterschiedlichen Versorgungs-, aber auch Sicherungsbedürfnissen der keineswegs homogenen Gruppe lebensälterer Strafgefangener Rechnung getragen werden kann. Unabhängig vom gewählten Behandlungsmodell kann die Pflege lebensälterer Strafgefangener ebenso wie die sogleich anzusprechende Sterbebegleitung nicht nur durch speziell geschulte Vollzugsbedienstete, sondern auch durch externe Kräfte wie ambulante Hospizdienste erfolgen [6, 11, 12, 15].

## Das Gefängnis ist (k)ein Ort zum Sterben^4^

Die Vorstellung von einem natürlichen oder durch eigene oder gar fremde Hand herbeigeführten Lebensende im Strafvollzug ist zweifelsohne bedrückend, bedarf aber einer differenzierten Betrachtung. Ausgeklammert bleiben sollen im Folgenden Tötungen von (älteren) Gefangenen durch Mitgefangene. Wie bereits dargestellt, gehört der Schutz des Lebens von Gefangenen zu den elementaren Verpflichtungen der Vollzugsbehörden und -bediensteten.

Kommt es alters- oder krankheitsbedingt zum Tod, stehen die Fragen im Vordergrund, ob der Vollzug auf ein solches natürliches Sterben vorbereitet sein kann und ob ein würdevolles Lebensende unter den Bedingungen des Freiheitsentzugs überhaupt möglich ist. Manche Stimmen verneinen dies und postulieren unter Berufung auf die Rechtsprechung des Bundesverfassungsgerichts (BVerfG) zur Verfassungsmäßigkeit der lebenslangen Freiheitsstrafe [17] ein Menschenrecht auf ein Sterben in Freiheit [7, 18]. Den Tod im Gefängnis per se als Verletzung der Menschenwürde anzusehen und auf den Ausweg einer Strafvollstreckungsunterbrechung gemäß § 455 Strafprozessordnung (StPO) zu verweisen [7], erscheint jedoch aus mehreren Gründen zweifelhaft. So hat das BVerfG keineswegs ausgeschlossen, dass „eine lebenslange Freiheitsstrafe im Wortsinne ein Leben lang vollstreckt wird“, sondern eine Strafaussetzungspraxis beanstandet, welche die Chance, der Freiheit wieder teilhaftig zu werden, „auf einen von Siechtum und Todesnähe gekennzeichneten Lebensrest reduziert“ ([19]; kritisch aus vollzugsärztlicher Sicht Bausch-Hölterhoff [20, S. 97 f.]).

Zu kurz greift jedenfalls das Argument, bei sterbenden Gefangenen habe das Vollzugsziel der Resozialisierung jeglichen Sinn verloren [18]. Die Wiedereingliederung des Gefangenen ist in der Tat das zentrale Anliegen des Vollzugs, legitimiert ihn aber nicht. Andernfalls müssten auch nicht resozialisierungsbedürftige, sozial gut integrierte wie auch einer Resozialisierung nicht zugängliche Täter entlassen werden. Dass Gefangene mit Freiheitsentzug bestraft werden, geschieht nicht primär um ihrer Resozialisierung willen, sondern beruht auf der vom Gericht zum Ausgleich ihrer Schuld zugemessenen Strafe. Wie im Jugendstrafrecht gilt auch hier: „Wir bestrafen nicht (mehr), um zu resozialisieren, sondern, wenn wir schon bestrafen müssen, versuchen wir zu resozialisieren“ [21]. Auch kann nicht gesagt werden, dass der Strafvollzug als Ausdruck der Schuldvergeltung und in seiner mittelbaren generalpräventiven Funktion mit dem absehbaren Lebensende des Gefangenen jeglichen Sinn verliert.

Entscheidender ist jedoch, dass es nicht wenige Gefangene gibt, die für sich „draußen“ keinen vorzugswürdigen Sterbeort sehen, sondern lieber im Gefängnis wegen der dort bestehenden Kontakte und vertrauten Umgebung sterben möchten [1, 6, 18, 20, 22]. Ihnen das zu ermöglichen, kann – auch wenn man den zugrunde liegenden Perspektivverlust bedauern mag – schlechterdings nicht menschenwürdewidrig sein. Dagegen dürften Fälle von Gefangenen, bei denen trotz eines absehbaren Lebensendes noch Flucht- oder Missbrauchsgefahr in einem Ausmaß angenommen werden kann, das kein Sterben in Freiheit zulässt, seltener sein und jedenfalls kaum vorkommen, wenn die Sterbephase begonnen hat [1, 7, 18].

Selbstverständlich sollten Gefangene möglichst nicht im Gefängnis sterben müssen und es bestehen auch die rechtlichen Möglichkeiten, dies zu verhindern. Das nahe Lebensende kann ein besonderer Umstand gemäß § 57 Abs. 2 Nr. 2 StGB sein. Diese Vorschrift erlaubt es unter der weiteren Voraussetzung einer günstigen Risikobeurteilung des Gefangenen (Legalprognose), die Vollstreckung der Strafe ausnahmsweise bereits nach Verbüßung der Hälfte einer zeitigen Strafe zur Bewährung auszusetzen. Auf vollstreckungsrechtlicher Ebene kommt weiterhin eine Unterbrechung der Strafvollstreckung wegen Vollzugsuntauglichkeit nach § 455 Abs. 4 Nr. 2 StPO (krankheitsbedingte nahe Lebensgefahr durch Vollstreckung) oder nach § 455 Abs. 4 Nr. 3 StPO (keine Behandelbarkeit in der Anstalt) in Betracht.

Wie eine Entscheidung des BVerfG aus dem Jahr 2010 [23] zeigt, wird diese Option jedoch von den Vollstreckungsbehörden und Vollstreckungsgerichten ausgesprochen restriktiv gehandhabt (Kinzig [1, S. 1601 f.] nennt entsprechende Fälle aus dem Maßregelvollzug). Im Fall eines Gefangenen, der an einem inoperablen Bronchialkarzinom in einer palliativen Situation litt, war eine auch von der Anstaltsärztin „dringend“ befürwortete Strafunterbrechung mehrfach verweigert worden. Hier musste das BVerfG darauf hinweisen, dass die Durchsetzung des staatlichen Strafanspruchs dann ihre Grenzen findet, wenn der Strafgefangene todkrank ist und von ihm nur eine sehr eingeschränkte Gefahr erneuter Straftaten ausgeht. Im selben Jahr rügte das Oberlandesgericht (OLG) Celle [24] die Verweigerung einer Strafunterbrechung für einen unheilbar an Zungen- und Lungenkrebs erkrankten, auf einen Rollator angewiesenen Gefangenen, die die Strafvollstreckungsbehörde mit „eine(r) mittlere(n) Gefahr des Begehens neuer Sexualstraftaten“ begründet hatte.

Schon im Jahr 2006 hat das Hanseatische OLG [25] entschieden, dass ein Sterben in Würde eine Strafunterbrechung bei einem nur noch eingeschränkt gefährlichen Straftäter auch dann gebieten kann, wenn die Voraussetzungen des § 455 Abs. 4 StPO nicht erfüllt sind: „Je kürzer die voraussichtliche Lebenserwartung ist, desto stärker fällt das Interesse des Strafgefangenen, im Kreise seiner engsten Angehörigen in Freiheit zu sterben, ins Gewicht.“

Neben einer vorrangigen regulären Strafunterbrechung kann noch an eine Gnadenentscheidung nach § 452 StPO in Verbindung mit den Gnadenordnungen der nach Satz 2 regelmäßig zuständigen Länder gedacht werden. Schließlich bieten jedenfalls bei absehbar kurzer Lebenszeit auch die vollzugsöffnenden Maßnahmen wie ein Langzeitausgang (§ 53 Abs. 2 Nr. 3 StVollzG NRW) Gestaltungsmöglichkeiten für ein Sterben in Freiheit [7, 13].

## Sterbebegleitung und Patientenautonomie im Strafvollzug

Bleibt der sterbende Gefangene in der JVA, gilt für Behandlungsentscheidungen am Lebensende der gleiche Rechtsrahmen wie auch außerhalb von Gefängnismauern. Das bedeutet, dass der Gefangene einen Anspruch auf eine effektive Symptomkontrolle hat, die insbesondere eine indizierte Schmerzmedikation bis hin zu einer palliativen Sedierung umfassen kann und ggf. auch unter Inkaufnahme einer Todesbeschleunigung erfolgen darf [26]. Die Verpflichtung zur Gesundheitsfürsorge beinhaltet neben dieser herkömmlich als indirekt bezeichneten Form der Sterbehilfe auch alle nicht lebensverkürzenden Ausprägungen der Hilfe beim Sterben wie eine adäquate Körperpflege sowie religiösen, spirituellen, sozialen und emotionalen Beistand, der durch Vollzugsbedienstete, Angehörige, Ehrenamtliche, aber auch durch Mitgefangene geleistet werden kann [6, 7, 15, 18].

Es ist offensichtlich, dass eine solche Sterbebegleitung unter normalen Vollzugsbedingungen in einer JVA kaum möglich ist, sondern die oben bereits angesprochene Verlegung in ein dafür ausgestattetes JVK oder eine externe Palliativstation erfordert. Bei medizinisch weniger anspruchsvoller Sterbebegleitung ist indes auch eine Verlegung in die Krankenabteilung der JVA denkbar, während ein würdiges begleitetes Sterben im Haftraum ein extremer Ausnahmefall sein dürfte.

Die Probleme liegen hier also wiederum nicht primär im rechtlichen Bereich, sondern faktisch vor allem darin, im Vollzug Rahmenbedingungen für ein menschenwürdiges Sterben zu schaffen, namentlich und auch im wahrsten Sinne des Wortes geeignete Sterberäume [18] bereitzustellen wie auch Sterberituale zu entwickeln [16]. Dass der institutionell nicht auf das Sterben vorbereitete Vollzug [7, 11, 12, 17, 18] in dieser Hinsicht erst am Anfang steht, machen Initiativen wie die Arbeitsgruppe „Begleitung Schwerkranker und Sterbender“ in der JVA Hövelhof [27] oder die im JVK Wittlich angeschaffte „Materialkiste“ zur Ausstattung von Sterbezimmern deutlich [28].

Selbstbestimmt handelnde Kranke haben das Recht, indizierte Maßnahmen der Lebensverlängerung und -erhaltung abzulehnen. Ebenso wie die Verpflichtung zur angemessenen Sterbebegleitung unterliegt dieses Recht keinen vollzuglichen Einschränkungen. Das Grundrecht auf selbstbestimmtes Sterben umfasst unstreitig die Patientenautonomie; dies ist im Vollzug in gleicher Weise zu respektieren wie auch außerhalb [29]. Zwar sehen alle Länderstrafvollzugsgesetze Ermächtigungen zu Zwangsmaßnahmen auf dem Gebiet der Gesundheitsfürsorge vor (§ 78 StVollzG NRW); diese Vorschriften betreffen jedoch nur Fälle krankheitsbedingt fehlender Einsicht in die Behandlungsnotwendigkeit oder die Abwehr von Fremdgefährdungen und lassen somit das Grundrecht auf autonomes Sterben unberührt.

Dies zeigt auch der von Lindemann und Verrel [8] mitgeteilte Erlass des nordrhein-westfälischen Ministeriums der Justiz zum erlaubten Sterbefasten im Strafvollzug und die Entscheidung des BVerfG [30] zum Verbot einer Zwangsbehandlung im Maßregelvollzug, die allein dem Schutz des Untergebrachten dient, die dieser aber wirksam ausgeschlossen hat.

Es ist folglich nicht nur der vom aufgeklärten einwilligungsfähigen Strafgefangenen aktuell geäußerte (Nicht‑)Behandlungswille zu respektieren. Im Fall fehlender Äußerungsfähigkeit entfaltet auch eine Patientenverfügung Verbindlichkeit, die § 1827 Abs. 1 Bürgerliches Gesetzbuch (BGB) entspricht; sollte eine solche nicht vorhanden sein, sind frühere Behandlungswünsche und der mutmaßliche Willen des Gefangenen zu beachten (§ 1827 Abs. 2 BGB).

In gleicher Weise gelten für Gefangene die betreuungsrechtlichen Regeln über die gesetzliche oder gewillkürte Vertretung entscheidungsunfähiger Patienten (§ 1814 BGB). Unterschiede dürften wiederum insoweit bestehen, als bei Gefangenen noch weniger Kenntnis über die bestehenden Vorsorgemöglichkeiten, geschweige denn deren tatsächlicher Gebrauch, vorausgesetzt werden kann als bei Patient*innen in Freiheit. Ist die Beschäftigung mit dem eigenen Tod schon ganz allgemein ein gesellschaftliches Tabu, gilt dies erst recht im Strafvollzug [6, 7, 31]. Umso mehr besteht die Notwendigkeit, die Möglichkeiten einer gesundheitlichen Versorgungsplanung auch in Justizvollzugsanstalten rechtzeitig anzusprechen und zu nutzen [18].

## Strafbarkeit der Tötung auf Verlangen

So wenig im Strafvollzug rechtliche Besonderheiten für den Umfang einer medizinischen Behandlung am Lebensende gelten, so unberührt bleibt auch das in § 216 StGB enthaltene Verbot einer vom Gefangenen gewünschten Tötung durch fremde Hand. Im Unterschied zur Herbeiführung des Todes durch eine erlaubte Behandlungsbegrenzung oder durch eine indizierte Symptomkontrolle wird der Tod hier durch einen nicht indizierten Eingriff bewirkt, der eine von der Grunderkrankung unabhängige neue Todesursache setzt [32].

Zwar hat der Bundesgerichtshof vor Kurzem angedeutet, § 216 StGB in solchen Extremfällen restriktiv auszulegen, in denen es dem Sterbewilligen faktisch unmöglich ist, sein Leben durch eigene Hand zu beenden und er daher zur Umsetzung seines Sterbewunsches auf fremde Hilfe angewiesen ist [33]. Derartige Fälle letztlich vollständig bewegungsunfähiger Kranker, deren Sterbewunsch auch nicht durch eine Therapiebegrenzung erfüllt werden kann, sind aber im Vollzug noch weniger vorstellbar als außerhalb. Es besteht daher auch und gerade im Strafvollzug kein Anlass, die Strafbarkeit der Tötung auf Verlangen zu relativieren (zur abweichenden Rechtslage in den Niederlanden Mevis [34, S. 108 ff.]).

## Suizid und Suizidhilfe im Strafvollzug

Die hohe Suizidrate unter Strafgefangenen [5, 8] gehört zu den besonders dunklen Seiten des strafenden Freiheitsentzugs. Gefangenensuizide sind für Vollzugsbedienstete wie Mitgefangene gleichermaßen bedrückende, unter Umständen sogar traumatisierende Ereignisse [5, 28] und zeigen, dass der vollzuglichen Suizidprävention eine herausragende Bedeutung zukommt. Die empirischen Grundlagen dafür sind gelegt [35, 36], es existieren bereits einzelne Konzepte [37, 38] und alle Landesstrafvollzugsgesetze geben die Befugnis, bei der Gefahr von Selbsttötungen Sicherungsmaßnahmen anzuordnen (§ 69 Abs. 1 StVollzG NRW). Manche lassen darüber hinaus ausdrücklich medizinische Zwangsmaßnahmen zu, „um den Erfolg eines Suizidversuchs zu verhindern“ (z. B. § 93 Abs. 1 Satz 1 des Niedersächsischen Strafvollzugsgesetzes).

Der bisherige Blick auf den Gefangenensuizid als ein unbedingt und mit allem Nachdruck zu verhinderndes Ereignis muss jedoch relativiert werden: Im Jahr 2020 erkannte das BVerfG [39] das Grundrecht auf autonomes Sterben, *einschließlich* der freiverantwortlichen Selbsttötung und Inanspruchnahme dazu angebotener Hilfe, an. Jedoch darf die Bedeutung dieser Entscheidung für den Strafvollzug nicht überschätzt werden, da nur freiverantwortlich gefasste Selbsttötungsentschlüsse grundrechtlich geschützt sind. Voraussetzung hierfür ist die zweifelsfreie Feststellung eines autonom, d. h. vor allem unbeeinflusst von einer akuten psychischen Störung, ohne unzulässigen Druck und mit einer gewissen Dauerhaftigkeit und inneren Festigkeit gefassten Suizidentschlusses [39, Rn. 240 ff.]. An diesem Erfordernis wird es gerade bei Gefangenen noch viel häufiger fehlen als bei in Freiheit lebenden Sterbewilligen.

Per se ausgeschlossen sind freiverantwortliche und auch als solche erkennbare Suizidentscheidungen im Strafvollzug gleichwohl und gerade bei lebensälteren Strafgefangenen nicht. So können letal erkrankte Gefangene ihren Sterbeprozess ohne Inanspruchnahme palliativmedizinischer Behandlung bis zum Schluss ebenso abkürzen wollen wie Patient*innen draußen. Es ist denkbar, dass in Gefangenen auch aus anderen Gründen, etwa weil ihnen eine realistische Entlassungsperspektive fehlt, ein wohl überlegter Entschluss zur Lebensbeendigung reift.

Das BVerfG hat sich in der Folgezeit in 2 Entscheidungen mit Verfassungsbeschwerden Gefangener befasst, deren Anträge auf Bereitstellung von Suizidmitteln durch die JVA abgelehnt wurden und die sich auf das Grundrecht auf autonomes Sterben berufen haben.

Im ersten Fall [40] eines seit über 35 Jahren aufgrund von 2 lebenslangen Freiheitstrafen in einer nordrhein-westfälischen JVA inhaftierten Gefangenen rügte das BVerfG die unzureichende Aufklärung des Sachverhalts durch die Gerichte. Außerdem äußerte es Zweifel daran, dass sich Vollzugsbedienstete auf eine Gewissensentscheidung berufen können. Das BVerfG hat sich aber nicht näher dazu verhalten, ob auch im Vollzug ein Grundrecht auf freiverantwortliche Selbsttötung besteht.

Im zweiten, Anfang 2025 entschiedenen Fall [41] wurde die Verfassungsbeschwerde eines in Hamburg zu 12 Jahren und 6 Monaten Freiheitsstrafe mit anschließender Sicherungsverwahrung verurteilten Gefangenen zwar nicht zur Entscheidung angenommen, da der Gefangene den Sachverhalt und die geltend gemachte Grundrechtsverletzung nicht substantiiert und schlüssig dargelegt hatte. Gleichwohl führt das BVerfG nach Betonung der staatlichen Lebensschutzpflicht gerade gegenüber der besonders vulnerablen Gruppe der Gefangenen aus, dass es mit den „… verfassungsrechtlichen Wertungen nicht vereinbar sein [dürfte], wenn der Strafvollzug dem Einzelnen für einen ersthaften, dauerhaften und freiverantwortlichen Suizid überhaupt keinen Raum gewährt und ihm somit faktisch nur die Möglichkeit eines sogenannten Brutalsuizids lässt“ ([41], Rn 26 unter vorhergehendem Verweis auf Verrel; [42] und Lindemann; [43]).

Damit sollte nicht länger zweifelhaft sein, dass das Grundrecht auf selbstbestimmtes Sterben im Strafvollzug nicht aus Gründen jedweder Strafzwecke, sondern nur wegen der vollzugsspezifischen Gefahren unfreier Suizidentschlüsse eingeschränkt werden kann (anderer Auffassung Stahlhacke [28, S. 207 ff.]). Solche Schranken können beispielsweise in besonderen Anforderungen an die Prüfung und Feststellung eines freien Suizidentschlusses bestehen. So reicht ein Sterbewunsch eines Gefangenen nicht aus, „der unter bloßem Verweis auf sein vergleichsweise hohes Alter und eine als perspektivlos empfundene Haftsituation“ [41, Rn 29] geäußert wird.

Es soll nicht verkannt werden, welche enormen Herausforderungen für den Vollzug mit der Respektierung eines im Einzelfall als autonom angesehenen Suizidwunsches verbunden sind. Hier wäre neben den Anforderungen an die Feststellung von Freiverantwortlichkeit etwa zu klären, ob Suizidassistenz im oder nur außerhalb des Vollzugs stattfinden darf, welche Rolle Anstaltsärzt*innen und Vollzugsbedienstete übernehmen dürfen [13, 18, 28], ob Angehörige anwesend sein dürfen, wie Mitgefangene Abschied nehmen können [7], wer die Kosten trägt, ob eine Obduktion erfolgen muss (einen umfangreichen Regelungsvorschlag unterbreitet Stahlhacke [28, S. 300 ff.]).

Diese Regelungsfragen gehen weit über das hinaus, was Gegenstand der bislang vorgelegten Gesetzentwürfe zur Neuregelung der Hilfe zur Selbsttötung ist.^5^ Ob es zu einer entsprechenden Regelung überhaupt kommt und welchen Inhalt diese bejahendenfalls hätte, ist derzeit nicht abzusehen. Erst recht ist nicht vorhersagbar, ob in einem weiteren Schritt Reformen in den Länderstrafvollzugsgesetzen erforderlich würden, um angemessen mit dem Ausnahmefall eines freiverantwortlichen Suizidwunsches Gefangener umzugehen (zur fortgeschritteneren Rechtsentwicklung in der Schweiz s. Tag [44]). Die grundrechtliche Absicherung freiverantwortlicher Gefangenensuizide führt allerdings schon jetzt dazu, dass die oben angesprochenen Zwangsmaßnahmen zur Abwendung von Suiziden nur bei unfreien Selbsttötungsentschlüssen ergriffen werden dürfen [42, 43].

## Fazit

„Der Justizvollzug ist ein Spiegelbild der Gesellschaft – wobei das Spiegelbild eher wie ein Brennglas wirkt.“ Diese kürzlich geäußerte Sicht des Leiters der Abteilung Justizvollzug im Justizministerium Baden-Württemberg [45] trifft auch auf die mit dem zunehmenden Alter von Gefangenen verbundenen Probleme zu. Der Vollzug ist mancherorts zwar auf einem guten Weg, „altengerecht“ zu werden, doch strukturell und traditionell in einer deutlich schlechteren Ausgangslage als die ebenfalls ausbaubedürftige Versorgung alter Menschen in Freiheit. Es wird künftig mehr Vollzugsbedienstete mit einer Ausbildung in Altenpflege und jedenfalls in den JVK eine ausreichende Bettenzahl geben müssen für Gefangene, die einer geriatrischen oder palliativmedizinischen Versorgung bedürfen.

Das Gefängnis muss kein schlechter Ort zum Sterben sein, doch gibt das Thema „Altern und Sterben im Strafvollzug“ allen Anlass dazu, sich der Ultima-Ratio-Funktion der Freiheitsstrafe bewusst zu werden, mit Nachdruck für eine Zurückdrängung und Begrenzung vollstreckter Freiheitsstrafen auf das wirklich unerlässliche Maß einzutreten und bei den Staatsanwaltschaften als Vollstreckungsbehörden einen grundrechtssensibleren Umgang mit § 455 StPO (Unterbrechung der Strafvollstreckung wegen Vollzugsuntauglichkeit) anzumahnen.

## Footnotes

1 Der Justizvollzug umfasst sowohl den in Strafanstalten erfolgenden Vollzug von Freiheitsstrafen als auch den in davon räumlich getrennten Einrichtungen stattfindenden Vollzug der Sicherungsverwahrung im Anschluss an eine Strafhaft. Soweit nicht ausdrücklich danach unterschieden wird, sind mit den Begriffen Strafvollzug, Strafgefangene und Gefängnis beide Vollzugsarten gemeint. Zu den hier nicht behandelten Besonderheiten des psychiatrischen Maßregelvollzugs (s. Kinzig [1, S. 1610 ff.]).

2 Frauen bilden in Justizvollzugsanstalten eine Minderheit von ca. 6 %. Es erscheint auch von daher vertretbar, im Folgenden nur die Bezeichnung für männliche Gefangene zu verwenden, obwohl damit Gefangene beiderlei Geschlechts gemeint sind.

3 https://search.coe.int/cm?i=0900001680a346cb; zugegriffen am 29.07.2025.

4 In Anlehnung an Bereswill und Neuber [16].

5 Bundestags-Drucksache 20/904 und 20/7624; s. zur bislang gescheiterten Neuregelung https://www.bundestag.de/dokumente/textarchiv/2023/kw27-de-suiziddebatte-954918 (Zugegriffen: 29. Juli 2025).
